# Interleukin-10 Promoter Gene Polymorphisms and Susceptibility to Tuberculosis: A Meta-Analysis

**DOI:** 10.1371/journal.pone.0127496

**Published:** 2015-06-01

**Authors:** Xuan Gao, Junjun Chen, Zhongkai Tong, Guangdie Yang, Yinan Yao, Fei Xu, Jianying Zhou

**Affiliations:** Department of Respiratory Diseases, First Affiliated Hospital of Zhejiang University School of Medicine, Zhejiang University, P.R. China; Fudan University, CHINA

## Abstract

**Objective:**

As an update to other recent meta-analyses, the purpose of this study was to explore whether interleukin-10 (IL-10) polymorphisms and their haplotypes contribute to tuberculosis (TB) susceptibility.

**Methods:**

We searched for published case-control studies examining IL-10 polymorphisms and TB in PubMed, EMBASE, Cochrane Central Register of Controlled Trials (CENTRAL), Wanfang databases and the Chinese National Knowledge Infrastructure (CNKI). Odds ratios (ORs) with 95% confidence intervals (CIs) were used to calculate the strengths of the associations.

**Results:**

A total of 28 studies comprising 8,242 TB patients and 9,666 controls were included in the present study. There were no significant associations between the -1082G/A, -819C/T, and -592A/C polymorphisms and TB in the pooled samples. Subgroup analyses revealed that the -819T allele was associated with an increased TB risk in Asians in all genetic models (T vs. C: OR=1.17, 95% CI=1.05-1.29, P=0.003; TT vs. CC: OR=1.37, 95% CI=1.09-1.72, P=0.006; CT+TT vs. CC: OR=1.33, 95% CI=1.09-1.63, P=0.006; TT vs. CT+CC: OR=1.17, 95% CI=1.02-1.35, P=0.03) and that the -592A/C polymorphism was significantly associated with TB in Europeans under two genetic models (A vs. C: OR=0.77, 95% CI=0.60-0.98, P=0.03; AA vs. CC: OR=0.53, 95% CI=0.30-0.95, P=0.03). Furthermore, the GCC IL-10 promoter haplotype was associated with an increased risk of TB (GCC vs. others: P=1.42, 95% CI=1.02-1.97, P=0.04). Subgroup analyses based on ethnicity revealed that the GCC haplotype was associated with a higher risk of TB in Europeans, whereas the ACC haplotype was associated with a lower TB risk in both Asians and Europeans.

**Conclusions:**

This meta-analysis suggests that the IL-10-819T/C polymorphism is associated with the risk of TB in Asians and that the IL-10-592A/C polymorphism may be a risk factor for TB in Europeans. Furthermore, these data indicate that IL-10 promoter haplotypes play a vital role in the susceptibility to or protection against the development of TB.

## Introduction

TB, an infectious disease primarily caused by *Mycobacterium tuberculosis* (*M*. *tuberculosis*), is a growing global public health problem. According to the World Health Organization, approximately one-third of the world’s population is infected with *M*. *tuberculosis*, though only 10% of individuals who are infected by the pathogen will develop clinical disease [1]. These data suggest that, in addition to *M*. *tuberculosis* itself, the development of TB after infection may also involve certain host factors, such as host immunity and genetics [2].

IL-10, which is expressed by activated monocytes/macrophages, natural killer (NK) cells, dendritic cells (DCs), mast cells, B cells, and regulatory T cell subsets, is known to have macrophage-deactivating properties and undermines the Th1-driven pro-inflammatory response by down-regulating the production of several cytokines. O'Leary et al. demonstrated that in macrophages, IL-10 may prevent phagosome maturation, thus leading to *M*. *tuberculosis* persistence in humans [3]. Several studies have also reported high levels of IL-10 production in TB patients [4,5]. Furthermore, in mouse models, over-expression of IL-10 may affect the recurrence of latent TB but shows little effect on susceptibility to primary infection [6]. These results indicate that the IL-10 gene and its gene product, IL-10, play a critical role in susceptibility to and pathogenesis of TB.

The IL-10 gene maps to chromosome 1q31-32. The IL-10 promoter is highly polymorphic, and three single nucleotide polymorphisms (SNPs) at positions -1082, -819, and -592 within the promoter region have been shown to correlate with IL-10 production [7]. Meanwhile, these polymorphisms exhibit strong linkage disequilibrium. Previous in vitro studies showed that the GCC haplotype of peripheral blood mononuclear cells was related to abundant IL-10 production, whereas the ATA haplotype was correlated with low levels of IL-10 production [7–11].

To date, many genetic epidemiology studies have assessed the association between IL-10 gene polymorphisms and the risk of TB in different populations [2,12–38]. However, the results from these studies were often inconsistent and inconclusive. This inconsistency may derive from a number of issues, including false-positive errors, lack of power, and minor impacts of IL-10 gene polymorphisms on TB susceptibility [39]. A meta-analysis is defined as research that analyzes previous research. Hence, results from previously published studies are gathered and statistically analyzed [40]. The purpose of the present study was to identify patterns among variant results, to find the sources of any inconsistencies among those results, and to eliminate the effects of random errors that are responsible for false-positive or false-negative interactions. Although there are already three published meta-analyses on these polymorphisms [41,42,43], confusing results remain unresolved. Furthermore, two of the previous studies failed to test the Hardy-Weinberg equilibrium (HWE). Liang B. et al. considered the HWE but still included those studies that were not consistent with the HWE [43]. Deviation from the HWE among the controls implies either a potential bias during control selection or genotyping errors. Moreover, Liang B. et al. missed four studies [29, 33–34, 36] and also incorporated repeated articles into their meta-analysis, such as Ansari A. et al. (2009) and Ansari A. et al. (2011) and Selvaraj P. et al. (2008) and Prabhu Anand S. et al. (2007). Therefore, we performed a meta-analysis of all eligible studies to derive a more precise estimation of the associations between IL-10 polymorphisms and TB risk.

## Methods

### Publication search

An elaborate search was conducted for studies that examined the association between IL-10 polymorphisms and TB [40]. Two independent reviewers (Gao and Chen) searched PUBMED, EMBASE, Cochrane Central Register of Controlled Trials (CENTRAL), Wanfang databases and the Chinese National Knowledge Infrastructure (CNKI) to identify available studies that were published by August 2014 [40]. The heading (MeSH) terms and/or text words used were as follows: ‘tuberculosis or *Mycobacterium tuberculosis*’ in combination with ‘interleukin 10 or interleukin-10 or IL-10 or IL 10’ and ‘polymorphism or variant or genetic or SNP’. We also perused the reference lists of all retrieved articles and relevant reviews. If the full text article could not be obtained from the databases, we tried to contact the authors. There were no restrictions placed on language, race, ethnicity or geographic area [43].

### Study selection and data extraction

Studies were included in this meta-analysis if they met the following criteria: (1) studies that evaluated IL-10 gene polymorphisms and TB risk; (2) case-control studies; (3) studies that provided sufficient data to calculate an OR and a 95% CI. Studies were excluded if they (1) contained overlapping data; (2) were based on families; (3) did not provide the numbers of null and wild-type genotypes or alleles; (4) were editorials, reviews, or abstracts; or (5) were not consistent with HWE.

Data were extracted from original studies independently by two reviewers (Gao and Chen). Any discrepancies between the reviewers were resolved either by reaching a consensus or by a third reviewer (Yao). The following information was collected from each study: the name of the first author, the year of publication, the originating country, ethnicity, types of TB infection and controls, human immunodeficiency virus (HIV) status, the number of cases and controls, and genotype and allele frequency information. We verified the accuracy of the data by comparing the collection forms from each investigator.

### Statistical analysis

When data from at least 5 similar studies were available, meta-analysis was performed. The summary ORs and 95% CIs were used to measure the strength of the associations between IL-10 polymorphisms and TB susceptibility [39]. The statistical significance of the summary ORs was evaluated using the Z test. For each SNP, we established four genetic models to evaluate their association with TB risk: (1) allelic contrast; (2) variant homozygote genotype vs. wild-type homozygote genotype; (3) dominant model: variant homozygote combined with a heterozygote genotype versus wild-type homozygote genotype, and (4) recessive model: variant homozygote genotype versus heterozygote and wild-type homozygote genotypes.

The heterogeneity between studies was assessed using the chi-square-based Cochrane Q-test, which was considered to be significant when P<0.10 [44]. The fixed-effect model shows that the similar impact of genetic factors on TB susceptibility among variant studies are purely accidental, whereas the random-effect model indicates that dramatic diversity in assessment exists due to both intra-study sampling errors and inter-study variances [40, 45]. The fixed-effect model was chosen when the P value from the chi-square test was greater than 0.10; otherwise, the random-effect model was used [46]. To explore the source of the heterogeneity and to evaluate ethnicity-specific effects, subgroup analyses performed for IL-10 polymorphisms were investigated in a sufficient number of studies. Publication bias was assessed by visual inspection of funnel plots, in which the standard error of the log (OR) of each study was plotted against the log (OR). Funnel plot asymmetry was assessed using Egger’s linear regression test [47]. Departure from HWE in the control group was assessed by the chi-square test, and a P-value <0.05 was considered significant.

All statistical tests were performed using Review manager 5.2 (Nordic Cochrane Center, Copenhagen, Denmark) and STATA 12.0 (Stata Corporation, College Station, TX) software. P values <0.05 were considered statistically significant.

## Results

### Characteristics of included studies

**Fig 1:**
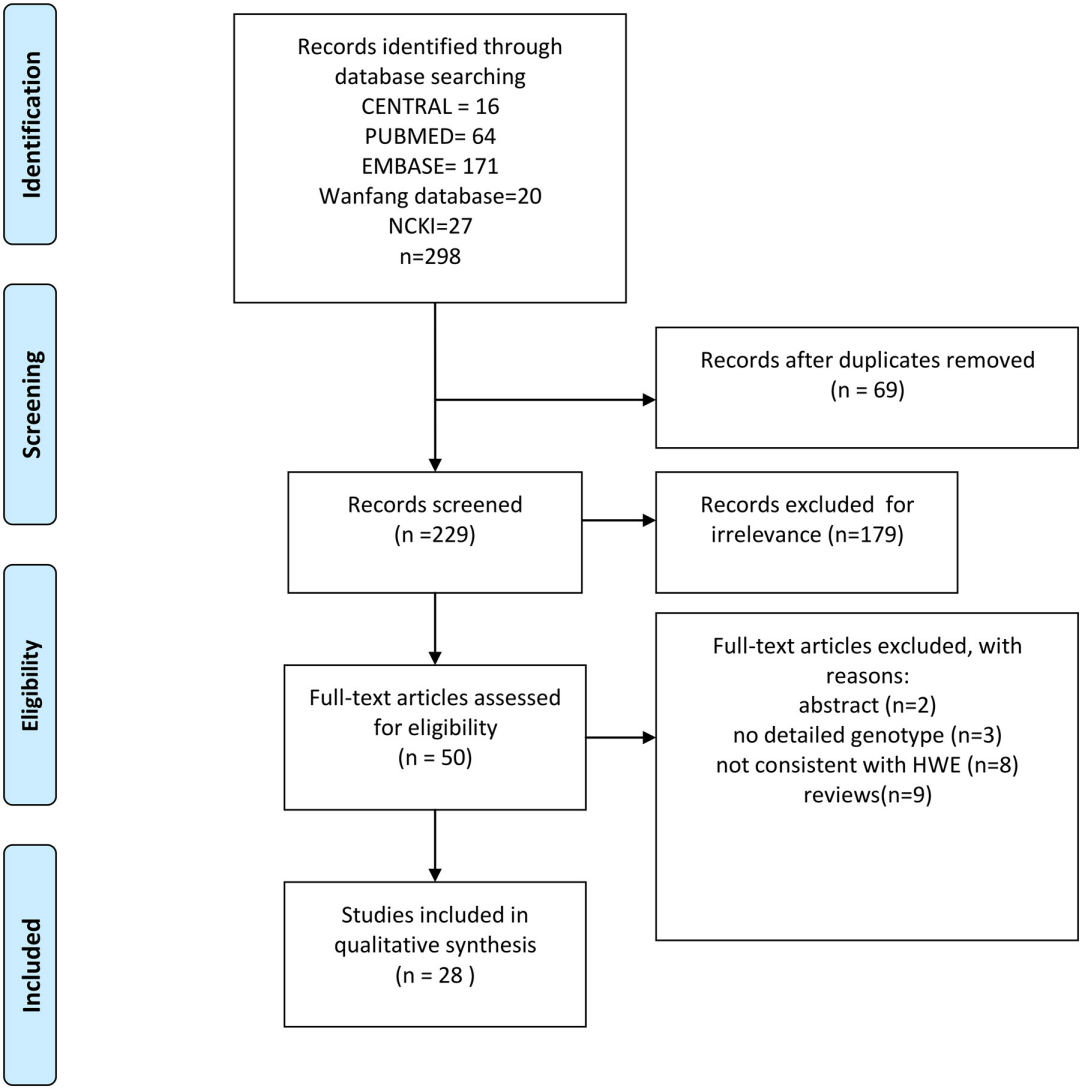
Flow chart depicting the study selection process.

The selection process of this literature review is summarized in the flow diagram (Fig 1). A total of 28 eligible articles fully met the inclusion criteria and were incorporated into this meta-analysis [2,12–38]. Of these studies, five were performed in Europeans, five in Africans, three in Americans and 15 in Asians. Table 1 shows the characteristics of these studies, and Table 2 provides the detailed genotype frequencies and the HWE assessment results.

**Table 1:** Characteristics of the case-control studies included in the meta-analysis.

Study [Ref]	Year	Country	Ethnicity	Type of infection	Type of controls	Cases(n)	Controls(n)	HIV status	SNPs
Bellamy [12]	1998	Gambia	African	Pulmonary TB	male donors	401	408	Negative	-1082G/A,-819C/T,-592C/A
Lopez-Maderuelo[13]	2003	Spain	European	Pulmonary TB	healthy tuberculin-negative volunteers	113	100	Negative	-1082G/A
Fitness [14]	2004	Malawi	African	TB	individually matched controls	514	913	Positive in 50% of cases and negative in control	-1082G/A,-819C/T
Shin [15]	2005	Korea	Asian	Pulmonary TB	healthy controls	459	871	Negative	-1082G/A,-819C/T,-592C/A,Haplotype
Tso [2]	2005	China	Asian	Pulmonary and extrapulmonary TB	healthy donors	385	471	Negative	Haplotype
Amirzargar [16]	2006	Iran	Asian	Pulmonary TB	healthy donors	41	123	NA	-819C/T,-592C/A
Oral [16]	2006	Turkey	European	Pulmonary, or pleural, other extrapulmonary TB	healthy donors	81	50	NA	-1082G/A,-819C/T,-592C/A,Haplotype
Ma [19]	2007	China	Asian	Pulmonary TB	healthy controls	40	40	NA	-1082G/A
Oh [18]	2007	Korea	Asian	Pulmonary TB	healthy adults	145	117	Negative	-1082G/A
Ates [20]	2008	Turkey	European	Pulmonary and extrapulmonary TB	healthy individuals	128	80	NA	-1082G/A,-819C/T,-592C/A,Haplotype
Selvaraj [21]	2008	India	Asian	Pulmonary TB	healthy subjects	166	188	Negative	-1082G/A,-819C/T
Wu [22]	2008	China	Asian	Pulmonary TB	miners with no TB	61	122	NA	-1082G/A,-819C/T,-592C/A,Haplotype
Moller [23]	2009	SouthAfrica	African	TB	healthy individuals with no TB	432	482	Negative	-1082G/A,-819C/T,-592C/A,Haplotype
Thye [24]	2009	Ghana	African	Pulmonary TB	cases with no TB contact	2010	2346	Negative	-1082G/A,-819C/T,-592C/A
Trajkov [25]	2009	Macedonia	European	TB	healthy individuals	75	301	NA	-819C/T,-592C/A
Taype [26]	2010	Peru	American	Pulmonary, or pleural, miliary other extrapulmonary TB	healthy control	626	513	NA	-1082G/A,-592C/A
Yang [27]	2010	China	Asian	Pulmonary TB	healthy subjects	200	200	NA	-1082G/A
Akgunes [28]	2011	Turkey	European	Pulmonary TB	healthy donors	30	30	NA	-1082G/A,-819C/T,-592C/A
Ben-Selma [29]	2011	Tunisia	African	Pulmonary and extrapulmonary TB	healthy donors	131	95	Negative	-819C/T,-592C/A
Liang L [30]	2011	China	Asian	Pulmonary TB and TB pleurisy	no history of TB or pleural disease	235	78	Negative	-1082G/A,-819C/T,-592C/A,Haplotype
Ma Hui [32]	2012	China	Asian	Pulmonary TB	no TB contacts	109	314	NA	-1082G/A
Ma MJ [33]	2012	China	Asian	Pulmonary TB	no TB controls	923	1033	Negative	-1082G/A,-819C/T,-592C/A
Mei [34]	2012	China	Asian	Pulmonary TB	healthy donors	169	156	NA	-592C/A
Xin DS [35]	2012	China	Asian	Pulmonary TB	no TB history patients and healthy subjects	308	310	Negative	-1082G/A
Ramaseri [31]	2012	India	Asian	Pulmonary and extrapulmonary TB	healthy volunteers	224	107	Positive in 47% of cases and negative in control	-1082G/A,-819C/T
Garcia [36]	2013	Mexico	American	Pulmonary TB	donors and healthcare workers	98	60	Negative	-1082G/A
Meenakshi [37]	2013	India	Asian	TB	healthy subjects	100	100	NA	-1082G/A
Hutz MH [38]	2014	Paraguay	American	TB	healthy individuals with no TB	38	58	NA	-819C/T,-592C/A

TB = Tuberculosis, NA = data not available

**Table 2:** Distribution of IL-10 genotypes in patients and controls.

Studies	TB			Control			HWE
	11	12	22	11	12	22	P value
-1082G/A							
Akgunes [28]	6	9	15	0	13	17	0.130
Ates [20]	26	65	37	6	32	42	0.978
Bellamy [12]	51	185	165	45	184	179	0.824
Fitness [14]	40	143	142	87	251	203	0.524
Garcia [36]	60	29	9	31	25	4	0.768
Liang L [30]	0	28	207	0	9	69	0.589
Lopez-Maderuelo [13]	33	47	33	29	50	21	0.949
Ma Hui [32]	29	35	45	32	130	152	0.591
Ma MJ [33]	14	165	744	7	183	843	0.388
Ma ZM [19]	2	16	22	1	6	33	0.292
Meenakshi [37]	4	81	15	16	59	25	0.058
Moller [23]	39	199	194	53	202	227	0.426
Oh [18]	4	43	98	19	53	45	0.612
Oral [17]	10	41	30	5	13	32	0.060
Ramaseri [31]	12	62	136	2	43	57	0.057
Selvaraj [21]	5	42	102	6	69	108	0.204
Shin [15]	2	53	394	9	124	718	0.168
Taype [26]	22	187	414	10	153	347	0.142
Thye [24]	117	630	794	160	783	1025	0.542
Wu [22]	1	12	48	0	18	104	0.379
Xin DS [35]	248	55	5	249	60	1	0.185
Yang [27]	3	26	169	1	44	155	0.253
-819C/T							
Akgunes [28]	19	10	1	11	13	6	0.553
Amirzargar [16]	19	20	2	62	52	9	0.671
Ates [20]	63	58	7	36	36	8	0.819
Bellamy [12]	120	192	89	114	206	88	0.779
Ben-Selma [29]	55	65	11	43	42	10	0.957
Fitness [14]	178	220	60	287	303	108	0.062
Liang L [30]	22	90	123	12	31	35	0.253
Ma MJ [33]	58	256	229	61	253	230	0.491
Moller [23]	207	186	39	201	229	52	0.267
Oral [17]	48	23	10	24	19	7	0.320
Ramaseri [31]	39	117	62	28	55	24	0.760
Selvaraj [21]	24	86	45	45	82	56	0.174
Shin [15]	39	173	238	91	384	376	0.631
Thye [24]	514	763	267	665	942	365	0.329
Trajkov [25]	35	35	5	155	125	19	0.348
Wu [22]	3	34	24	10	62	50	0.125
Hutz MH [38]	0	6	32	0	7	51	0.625
-592A/C							
Akgunes [28]	1	10	19	6	14	10	0.785
Amirzargar [16]	2	20	18	9	52	62	0.671
Ates [20]	7	58	63	8	36	36	0.819
Bellamy [12]	89	192	120	88	206	114	0.779
Ben-Selma [29]	12	63	56	10	42	43	0.957
Liang L [30]	123	90	22	35	31	12	0.253
Ma MJ [33]	370	432	121	440	476	117	0.491
Mei [34]	56	81	32	26	79	51	0.622
Moller [23]	39	186	207	51	230	201	0.213
Oral [17]	10	23	48	7	19	24	0.320
Shin [15]	238	173	39	376	384	91	0.631
Taype [26]	117	218	264	105	230	178	0.055
Thye [24]	172	532	321	269	696	480	0.551
Trajkov [25]	5	31	39	28	117	154	0.403
Wu [22]	24	34	3	50	62	10	0.125
Hutz MH [38]	32	6	0	51	7	0	0.625

TB = Tuberculosis; HWE = Hardy-Weinberg equilibrium.

Twenty-two of the 28 articles studied the -1082G/A IL-10 polymorphism, 17 studied the -819C/T polymorphism, 16 studied the -592A/C polymorphism, and 6 studied IL-10 promoter haplotypes.

### The IL-10-1082G/A polymorphism is not associated with TB susceptibility

The associations between the -1082G/A polymorphism and TB are shown in Table 3. A total of 22 studies containing 6,699 TB patients and 7,679 controls were included in this meta-analysis. The results showed that the -1082G/A polymorphism was not associated with TB susceptibility under any genetic model. In addition, stratification by ethnicity revealed no association between the -1082G/A polymorphism and TB.

**Table 3:** Meta-analysis of the association between the IL-10–1082 G/A polymorphism and TB.

		A vs. G			AA vs. GG			AA+AG vs. GG			AA vs. AG+GG		
Population	No.	OR (95% CI)	*P*_*Eff*_	P_Het_	OR (95% CI)	*P*_*Eff*_	P_Het_	OR (95% CI)	*P*_*Eff*_	P_Het_	OR (95% CI)	*P*_*Eff*_	P_Het_
Overall	22	0.97(0.85–1.11)	0.67	<0.0001	0.88(0.63–1.24)	0.46	<0.0001	0.87(0.65–1.15)	0.32	<0.0001	1.00(0.84–1.19)	1.00	<0.0001
Subgroup by ethnicity													
Asian	12	1.07(0.82–1.38)	0.63	<0.0001	1.01(0.44–2.34)	0.98	<0.0001	0.93(0.48–1.82)	0.83	<0.0001	1.12(0.82–1.52)	0.48	<0.0001
European	4	0.62(0.36–1.07)	0.08	0.004	0.42(0.13–1.37)	0.15	0.008	0.55(0.24–1.26)	0.16	0.08	0.61(0.28–1.34)	0.22	0.003
African	4	1.01(0.91–1.11)	0.92	0.11	1.10(0.92–1.32)	0.30	0.26	1.11(0.93–1.32)	0.24	0.42	0.99(0.90–1.10)	0.88	0.23

TB = Tuberculosis; P_*Eff*_ = P value of pooled effect; P_*Het*_ = P value of heterogeneity test.

### Association between the IL-10-819C/T polymorphism and TB susceptibility

The survey results regarding the associations between the -819C/T polymorphism and TB are shown in Table 4. Our meta-analysis of the 17 case-control studies (5,024 TB patients and 6,180 controls) revealed that the -819C/T polymorphism was not associated with TB susceptibility under any genetic model.

**Table 4:** Meta-analysis of the association between the IL-10 -819C/T polymorphism and TB.

		T vs. C			TT vs. CC			CT+TT vs. CC			TT vs. CT+CC		
Population	No.	OR (95% CI)	*P*_*Eff*_	P_Het_	OR (95% CI)	*P*_*Eff*_	P_Het_	OR (95% CI)	*P*_*Eff*_	P_Het_	OR (95% CI)	*P*_*Eff*_	P_Het_
Overall	17	1.03(0.94–1.12)	0.57	0.04	1.01(0.89–1.14)	0.90	0.16	1.05(0.92–1.19)	0.46	0.09	1.01(0.92–1.11)	0.83	0.21
Subgroup by ethnicity													
Asian	7	1.17(1.05–1.29)	0.003	0.49	1.37(1.09–1.72)	0.006	0.67	1.33(1.09–1.63)	0.006	0.70	1.17(1.02–1.35)	0.03	0.32
European	4	0.77(0.52–1.15)	0.20	0.07	0.61(0.34–1.11)	0.11	0.24	0.85(0.62–1.16)	0.30	0.16	0.66(0.37–1.17)	0.15	0.36
African	5	0.97(0.90–1.04)	0.33	0.64	0.91(0.79–1.06)	0.22	0.89	0.98(0.89–1.09)	0.74	0.32	0.91(0.80–1.04)	0.16	0.87

TB = Tuberculosis; P_*Eff*_ = P value of pooled effect; P_*Het*_ = P value of heterogeneity test.

Subgroup analysis by ethnicity revealed that the -819T allele was associated with increased TB risk in Asians under all genetic models (T vs. C: OR = 1.17, 95% CI = 1.05–1.29, P = 0.003; TT vs. CC: OR = 1.37, 95% CI = 1.09–1.72, P = 0.006; CT+TT vs. CC: OR = 1.33, 95% CI = 1.09–1.63, P = 0.006; TT vs. CT+CC: OR = 1.17, 95% CI = 1.02–1.35, P = 0.03). However, no association was found in Europeans or Africans under any genetic model.

### Association of the IL-10-592A/C polymorphism with TB susceptibility

The results of our meta-analysis of the association between the -592A/C polymorphism and TB are shown in Table 5. A total of 16 case-control studies that examined the relationship between the -592A/C polymorphism and TB risk were included in this meta-analysis. The total number of cases and controls were 4,818 and 5,823, respectively. Meta-analysis revealed no remarkable association between the -592A/C polymorphism and TB in the selected samples.

**Table 5:** Meta-analysis of the association between the IL-10 -592A/C polymorphism and TB.

Population		A vs. C			AA vs. CC			AA vs. AC+CC			AA+AC vs. CC		
	No.	OR (95% CI)	*P*_*Eff*_	P_Het_	OR (95% CI)	*P*_*Eff*_	P_Het_	OR (95% CI)	*P*_*Eff*_	P_Het_	OR (95% CI)	*P*_*Eff*_	P_Het_
Overall	16	0.99(0.87–1.12)	0.84	<0.0001	0.99(0.79–1.25)	0.96	0.002	1.02(0.86–1.21)	0.79	0.006	1.02(0.86–1.20)	0.84	0.009
Subgroup by ethnicity													
Asian	6	1.22(0.96–1.55)	0.11	0.0004	1.50(0.89–2.53)	0.13	0.001	1.26(0.91–1.74)	0.17	0.002	1.32(0.94–1.85)	0.11	0.04
European	4	0.77(0.60–0.98)	0.03	0.16	0.53(0.30–0.95)	0.03	0.38	0.59(0.33–1.03)	0.06	0.50	0.77(0.56–1.05)	0.10	0.21
African	4	0.96(0.88–1.04)	0.39	0.48	0.92(0.77–1.10)	0.36	0.81	0.91(0.77–1.07)	0.25	0.84	0.98(0.86–1.11)	0.73	0.18

TB = Tuberculosis; P_*Eff*_ = P value of pooled effect; P_*Het*_ = P value of heterogeneity test.

However, after stratification by different ethnicities, a significant association was found in Europeans using two genetic models (A vs. C: OR = 0.77, 95% CI = 0.60–0.98, P = 0.03; AA vs. CC: OR = 0.53, 95% CI = 0.30–0.95, P = 0.03); this association was not observed in Asians or Africans under any genetic model.

### IL-10 promoter haplotype and TB

Three SNPs in the promoter region (-1082G/A, -819C/T, -592A/C) were in complete linkage disequilibrium, and three haplotypes exist (GCC, ACC, and ATA). Six of the eligible case-control studies analyzed the relationship between the IL-10 promoter haplotype and the risk of TB (Table 6). The results of pooling all studies demonstrated that the GCC haplotype was associated with an increased risk of TB (GCC vs. others: P = 1.42, 95% CI = 1.02–1.97, P = 0.04), but no association was found between the ACC and ATA haplotypes and TB risk. Furthermore, subgroup analyses based on ethnicity showed that the GCC haplotype was associated with an increased TB risk in Europeans, whereas the ACC haplotype was associated with a lower TB risk in both Asians and Europeans (Table 6).

**Table 6:** Meta-analysis of the association between IL-10 promoter haplotype (-1082G/A, 819C/T, 592A/C) and TB.

Population		GCC vs. others				ACC vs. others				ATA vs. others		
	No.	OR (95% CI)	*P*_*Eff*_	P_Het_	n	OR (95% CI)	*P*_*Eff*_	P_Het_	n	OR (95% CI)	*P*_*Eff*_	P_Het_
Overall	6	1.42(1.02–1.97)	0.04	0.009	6	0.85(0.68–1.05)	0.14	0.02	5	0.90(0.78–1.04)	0.14	0.74
Subgroup by ethnicity												
Asian	3	1.30(0.93–1.82)	0.12	0.70	2	0.84(0.72–0.99)	0.04	0.78	2	1.04(0.79–1.38)	0.76	0.87
European	2	2.14(1.53–3.01)	<0.0001	0.80	2	0.60(0.43–0.83)	0.002	0.39	2	0.78(0.56–1.09)	0.15	0.77

TB = Tuberculosis; P_*Eff*_ = P value of pooled effect; P_*Het*_ = P value of heterogeneity test.

### Heterogeneity and publication bias

**Fig 2:**
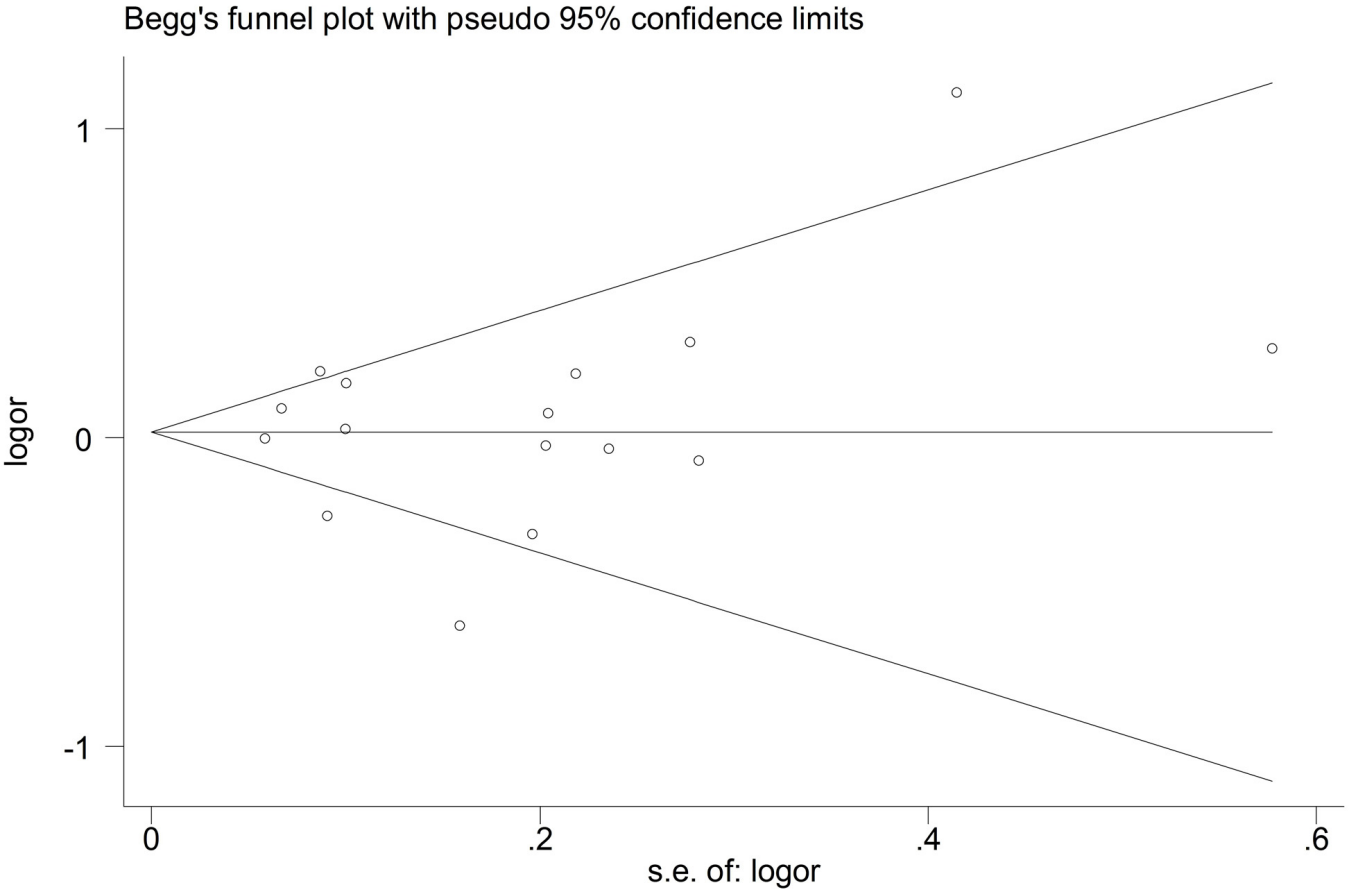
Funnel plot for -592A/C in the allele model.

Some intra-study heterogeneity was observed during the meta-analyses, but no evidence suggested heterogeneity between the significant associations, except for the GCC haplotype as a whole. This heterogeneity was eliminated after stratification by ethnicity. The funnel plots for these polymorphisms in all compared models were symmetrical (Fig 2 shows the funnel plot for -592A/C in the allele model). The results of the Egger’s test did not suggest obvious publication bias for the -819C/T variant (P = 0.711 for T vs. C, P = 0.949 for TT vs. CC, P = 0.533 for CT+TT vs. CC, P = 0.173 for TT vs. CT+CC). Similarly, no publication bias was detected for the associations between the -1082G/A and -592A/C polymorphisms and TB.

## Discussion

It is currently believed that host genetic factors are of vital importance in the pathogenesis of TB, as host genetic factors affect the expression levels of cytokines and chemokines that are known to participate in host immunity [48]. As a powerful Th2-regulatory cytokine, IL-10 plays an essential role during the latent stage of TB infection. The long arm of chromosome 1, where the IL-10 gene is situated, contains known polymorphisms within the IL-10 promoter region, including -1082G/A, -819T/C, and -592A/C [43]. Furthermore, IL-10 is reportedly associated with TB in different ethnic backgrounds [49].

The meta-analysis performed by Zhang J. et al. reported that the -1082G/A polymorphism correlated significantly with a downside risk of TB in Europeans, whereas the IL-10 -819T/C and -592A/C polymorphisms were unrelated to TB susceptibility [41]. Similarly, another meta-analysis by Liang B. et al. confirmed that the risk for TB was independent of the -1082G/A, -819T/C, and -592A/C genotypes in the gross population but showed that the risk was dramatically reduced in the -1082G/A genotype in Europeans and Americans and was significantly associated with the -819T/C polymorphism in Asians [43].

However, in our meta-analysis, no association was revealed between the IL-10-1082G/A, -819T/C and -592A/C polymorphisms and TB susceptibility from 22 studies with 6,699 TB patients and 7,679 controls, 17 studies with 5,024 TB patients and 6,180 controls, and 16 studies with 4,818 cases and 5,823 controls, respectively. According to our subgroup analyses by ethnicity, no association was revealed between the -1082G/A polymorphism and TB. Additionally, the -819T allele was found to be associated with an increased risk of TB in Asians under all genetic models, whereas two genetic models (A vs. C; AA vs. CC) of the association between the -592A/C polymorphism and TB showed significant associations in Europeans. Several reasons may explain why our results differ from those of Zhang J. et al. and Liang B. et al. First, we only incorporated studies that were consistent with HWE. Second, our work was an update to the work of other groups, which allowed for the inclusion of some new studies. As a result, our conclusions may be more scientific. Taken together, our results suggest that ethnic differences may play an important role in environmental and genetic factors.

We also analyzed the association between IL-10 promoter haplotypes and TB risk. In our meta-analysis of 6 studies, only the GCC haplotype was associated with an increased TB risk. Moreover, subgroup analyses based on ethnicity showed that the GCC haplotype was associated with an increased TB risk in Europeans, whereas the ACC haplotype was associated with a lower TB risk in both Asians and Europeans, suggesting that ethnic differences may play a role in the association between IL-10 promoter haplotypes and TB risk.

As an indispensable tool, genome-wide association studies (GWASs) are being used more and more for the identification of common variants that are associated with a variety of diseases. To date, many GWASs have successfully identified TB susceptibility genes [50–58]. These genes include the interferon-gamma gene (*IFNG*), the vitamin D receptor gene (*VDR*), and the interleukin-12 p40 subunit gene (*IL12B*), among others [52, 57]. However, these studies provided no direct evidence to prove an association between TB and the IL-10 gene. Furthermore, GWASs of TB are ongoing, which indicates that TB has not been adequately studied by modern genomic technologies [59]. Many of the associated genes have not yet been studied. With an increased number of GWASs studying TB, more related genes will be found, and IL-10 is only one gene. Therefore, with respect to genome-wide associations and TB, much work remains to be done.

Two of the selected studies in our meta-analysis considered the impact of HIV status on susceptibility to TB [14, 31]. However, many researchers have focused on this issue. Their studies have found that, compared to HIV-negative controls, HIV-positive patients showed greater susceptibility to TB [60]. Especially in sub-Saharan Africa, which has the highest HIV morbidity worldwide, HIV-positive persons showed a 20-fold increased risk over HIV-negative individuals of developing TB [61]. An increasing number of studies have begun to investigate the mechanism of how HIV infection influences susceptibility to TB. It was reported that antigens such as HLA-A31 and HLA-B41, chemokine receptors such as CCR5, and the -1082G allele of IL-10 were involved in TB susceptibility [31, 62–63]. However, the exact mechanism remains unclear, and additional studies are needed to clarify this issue.

Although some intra-study heterogeneity was detected for these polymorphisms during the meta-analyses, no evidence of heterogeneity was found for the significant associations. After subgroup analyses by ethnicity, the heterogeneity disappeared. This suggests that ethnicity may be the main source of heterogeneity. Furthermore, we generated funnel plots and carried out Egger’s tests to evaluate the existence of publication bias; no publication bias was observed in our study.

Several limitations should be considered when interpreting our results. First, additional studies are needed to complete a comprehensive analysis, especially for the IL-10 promoter haplotype [64]. Furthermore, after stratification by ethnicity, there were only a small number of studies in the European subgroup, which may reduce the strength of our conclusions. Second, different diagnostic criteria of TB and controls across studies may affect the comparability of the studies or lead to the misclassification of cases. The studies that were selected for this meta-analysis did not have unified diagnostic criteria, which may result in misclassification bias [59]. Third, the evaluation of our analysis is unadjusted. However, the accuracy of our evaluation with respect to the effects of gene-gene and gene-environment associations in TB has been compromised due to the limited amount of original data from the qualified studies [64].

In conclusion, this meta-analysis suggested that the IL-10-819T/C polymorphism was associated with TB risk in Asians and that the IL-10-592A/C polymorphism may be a risk factor for TB in Europeans. IL-10 promoter haplotypes play a vital role in the susceptibility to or protection against the development of TB. To further establish these associations, future studies with larger sample sizes and multi-ethnic sample groups are required.

## Supporting Information

S1 File Excluded articles with reasons. (DOCX)

S1 Meta-Analysis Checklist Meta analysis on genetic association studies form. (DOCX)

S1 PRISMA Checklist PRISMA 2009 Checklist. (DOC)

S1 Table Criteria for TB and controls in the case-control studies included in the meta-analysis. (DOCX)

S2 Table Meta-analysis of the association between the IL-10 -1082G/A polymorphism and TB for the random-effect model. (DOCX)

S3 Table Meta-analysis of the association between the IL-10 -1082G/A polymorphism and TB for the fixed-effect model. (DOCX)

S4 Table Meta-analysis of the association between the IL-10 -819C/T polymorphism and TB for the random-effect model. (DOCX)

S5 Table Meta-analysis of the association between the IL-10 -819C/T polymorphism and TB for the fixed-effect model. (DOCX)

S6 Table Meta-analysis of the association between the IL-10 -592A/C polymorphism and TB for the random-effect model. (DOCX)

S7 Table Meta-analysis of the association between the IL-10 -592A/C polymorphism and TB for the fixed-effect model. (DOCX)

S8 Table Meta-analysis of the association between IL-10 promoter haplotype (-1082G/A, 819C/T, 592A/C) and TB for the random-effect model. (DOCX)

S9 Table Meta-analysis of the association between IL-10 promoter haplotype (-1082G/A, 819C/T, 592A/C) and TB for the fixed-effect model. (DOCX)
