# Supplementary material for: Interleukin-10 Promoter Gene Polymorphisms and Susceptibility to Tuberculosis: A Meta-Analysis
Source: PLoS One. 2015 Jun 1;10(6):e0127496. doi: 10.1371/journal.pone.0127496 (PMC4452516; doi:10.1371/journal.pone.0127496)
Supplement: S1 File — (DOCX) [file pone.0127496.s001.docx]

Excluded Articles With Reasons

After screening and removing of duplicates and irrelevance, there were 50 studies full-text articles assessed for eligibility, and then 22 studies were excluded, with reasons as follows:

1. Reason: Articles which were not consistent with HWE

1.1. Ulger M, Emekdas G, Aslan G, Tas D, Ilvan A, et al. (2013) Determination of the cytokine gene polymorphism and genetic susceptibility in tuberculosis patients. Mikrobiyol Bul 47: 250-264.

1.2. Ansari A, Hasan Z, Dawood G, Hussain R (2011) Differential combination of cytokine and interferon-gamma +874 T/A polymorphisms determines disease severity in pulmonary tuberculosis. PLoS One 6: e27848.

1.3. Afzal MS, Anjum S, Salman A, Ashraf S, Farooqi ZUR, et al. (2011) Interleukin-10 gene promoter polymorphism as a potential host susceptibility factor in pakistani patients with pulmonary tuberculosis. Afr J Biotechnol. 10: 14706-14710.

1.4. Delgado JC, Baena A, Thim S, Goldfeld AE (2002) Ethnic-specific genetic associations with pulmonary tuberculosis. J Infect Dis 186: 1463-1468.

1.5. Mhmoud N, Fahal A, van de Sande WJ (2013) Association of IL-10 and CCL5 single nucleotide polymorphisms with tuberculosis in the Sudanese population. Trop Med Int Health 18: 1119-1127.

1.6. Mosaad YM, Soliman OE, Tawhid ZE, Sherif DM (2010) Interferon-gamma +874 T/A and interleukin-10 -1082 A/G single nucleotide polymorphism in Egyptian children with tuberculosis. Scand J Immunol 72: 358-364.

1.7. Scola L, Crivello A, Marino V, Gioia V, Serauto A, et al. (2003) IL-10 and TNF-alpha polymorphisms in a sample of Sicilian patients affected by tuberculosis: implication for ageing and life span expectancy. Mech Ageing Dev 124: 569-572.

1.8. Liu X, Sun Y, Guo M, Feng F (2009) Study on the relationship of interleukin-10 genetic polymorphisms with the susceptibility of pulmonary tuberculosis. Modern Preventive Medicine 36: 1827-1830.

2. Reason: Articles which were reviews

2.1. Leandro AC, Rocha MA, Cardoso CS, Bonecini-Almeida MG (2009) Genetic polymorphisms in vitamin D receptor, vitamin D-binding protein, Toll-like receptor 2, nitric oxide synthase 2, and interferon-gamma genes and its association with susceptibility to tuberculosis. Braz J Med Biol Res 42: 312-322.

2.2. Qidwai T, Jamal F, Khan MY (2012) DNA sequence variation and regulation of genes involved in pathogenesis of pulmonary tuberculosis. Scand J Immunol 75: 568-587.

2.3. Goldfeld AE (2004) Genetic susceptibility to pulmonary tuberculosis in Cambodia. Tuberculosis 84: 76-81.

2.4. Yim JJ, Selvaraj P (2010) Genetic susceptibility in tuberculosis. Respirology 15: 241-256.

2.5. Pacheco, A. G., Cardoso CC (2008) IFNG +874T/A, IL10 -1082G/A and TNF -308G/A polymorphisms in association with tuberculosis susceptibility: a meta-analysis study. Hum Genet 123: 477-484.

2.6. Zhang, J., Chen Y (2011) Interleukin-10 polymorphisms and tuberculosis susceptibility: a meta-analysis. Int J Tuberc Lung Dis 15: 594-601.

2.7. Lykouras D, Sampsonas F, Kaparianos A, Karkoulias K, Tsoukalas G, et al. (2008) Human genes in TB infection: their role in immune response. Monaldi Arch Chest Dis 69: 24-31.

2.8. Modlin RL, Bloom BR (2013) TB or not TB: That is no longer the question. Sci Transl Med 5.

2.9. Khalilullah SA, Harapan H, Hasan NA, Winardi W, Ichsan I, et al. (2014) Host genome polymorphisms and tuberculosis infection: What we have to say? Egyptian Journal of Chest Diseases and Tuberculosis 63: 173-185.

3. Reason: Articles which were conference papers

3.1. Druszczynska M, Wlodarczyk M, Slomczynska M, Janiszewska-Drobinska B, Trzepinski P, et al. (2012) Relationship of IL-10 gene polymorphism (-1082A/G) with cytokine production and tuberculosis (TB) suceptibility in Polish population. Immunology 137: 599.

3.2. Metanat M, NarooieNejad M, Sanei-Moghaddam E, Salehi M, Moazen J, et al. (2013) Comparison of gene polymorphism of TNF-(alpha) (G/A-308, G/A-238). Iran J Allergy Asthma Immunol 12: S70.

4. Reason: Articles which had no detailed genotype

4.1. Stein CM, Zalwango S, Chiunda AB, Millard C, Leontiev DV, et al. (2007) Linkage and association analysis of candidate genes for TB and TNF(alpha) cytokine expression: Evidence for association with IFNGR1, IL-10, and TNF receptor 1 genes. Hum Genet 121: 663-673.

4.2. Henao MI, Montes C, Paris SC, Garcia LF (2006) Cytokine gene polymorphisms in Colombian patients with different clinical presentations of tuberculosis. Tuberculosis (Edinb) 86: 11-19.

4.3. Tso, H. W., W. K. Ip (2005). Association of interferon gamma and interleukin 10 genes with tuberculosis in Hong Kong Chinese. Genes Immun 6: 358-363.
