# Supplementary material for: Interleukin-10 Promoter Gene Polymorphisms and Susceptibility to Tuberculosis: A Meta-Analysis
Source: PLoS One. 2015 Jun 1;10(6):e0127496. doi: 10.1371/journal.pone.0127496 (PMC4452516; doi:10.1371/journal.pone.0127496)
Supplement: S1 Table — (DOCX) [file pone.0127496.s004.docx]

**Table S1. Criteria of TB and controls in the case-control studies included in the meta-analysis.**

| Study[Ref] | Criteria |
| --- | --- |
| Bellamy[12] | AFB smear |
| Lopez-Maderuelo[13] | sputum culture-positive |
| Fitness[14] | culture, smear, or histology |
| Shin[15] | AFB |
| Tso[2] | culture, smear, clinical-radiological and histological evidences |
| Amirzargar[16] | AFB and CXR |
| Oral[16] | clinical, radiographic, pathologic and culture |
| Ma[19] | smear, culture, clinical and radiology |
| Oh[18] | smear, culture, clinical and radiology |
| Ates[20] | smear, culture, clinical and radiology |
| Selvaraj[21] | smear, culture, clinical and radiology |
| Wu[22] | sputum culture, X-ray and symptom |
| Moller[23] | bacteriological analyses |
| Thye[24] | smear, culture and X-ray |
| Trajkov[25] | WHO criteria |
| Taype[26] | NA |
| Yang[27] | CXR, smear and culture |
| Akgunes[28] | AFB smear |
| Ben-Selma[29] | ATS criteria |
| Liang L[30] | sputum smears and culture |
| Ma Hui[32] | smear and/or culture; X-ray and symptoms; follow-up |
| Ma MJ[33] | smear and/or culture; X-ray and symptoms; follow-up |
| Mei[34] | smear or/and X-ray |
| Xin DS[35] | smear or/and X-ray |
| Ramaseri[31] | smear and/or culture and CXR |
| Garcia[36] | history, isolation, signs and symptoms, radiograph |
| Meenakshi[37] | radiographic and histocytological examination, AFB |
| Hutz MH[38] | X-ray, contact history, and clinical symptoms |

TB=Tuberculosis, AFB=Acid-fast bacilli, CXR=Chest X Ray, WHO=World Health Organization, NA=data not available, ATS=American Thoracic Society.
