# Supplementary material for: Interleukin-10 Promoter Gene Polymorphisms and Susceptibility to Tuberculosis: A Meta-Analysis
Source: PLoS One. 2015 Jun 1;10(6):e0127496. doi: 10.1371/journal.pone.0127496 (PMC4452516; doi:10.1371/journal.pone.0127496)
Supplement: S2 Table — (DOCX) [file pone.0127496.s005.docx]

**Table S2. Meta-analysis of the association between the IL-10 -1082 G/A polymorphism and TB for random effect model.**

|  | No. | A vs G | | | AA vs GG | | | AA+AG vs GG | | | AA vs AG+GG | | |
| --- | --- | --- | --- | --- | --- | --- | --- | --- | --- | --- | --- | --- | --- |
| Population |  | OR(95% CI) | *P_Eff_* | P_Het_ | OR(95% CI) | *P_Eff_* | P_Het_ | OR(95% CI) | *P_Eff_* | P_Het_ | OR(95% CI) | *P_Eff_* | P_Het_ |
| Overall | 22 | 0.97(0.85-1.11) | 0.67 | <0.0001 | 0.88(0.63-1.24) | 0.46 | <0.0001 | 0.87(0.65-1.15) | 0.32 | <0.0001 | 1.00(0.84-1.19) | 1.00 | <0.0001 |
| Subgroup by ethnicity | | | | | | | | | | | | | |
| Asian | 12 | 1.07(0.82-1.38) | 0.63 | <0.0001 | 1.01(0.44-2.34) | 0.98 | <0.0001 | 0.93(0.48-1.82) | 0.83 | <0.0001 | 1.12(0.82-1.52) | 0.48 | <0.0001 |
| European | 4 | 0.62(0.36-1.07) | 0.08 | 0.004 | 0.42(0.13-1.37) | 0.15 | 0.008 | 0.55(0.24-1.26) | 0.16 | 0.08 | 0.61(0.28-1.34) | 0.22 | 0.003 |
| African | 4 | 1.01(0.91-1.11) | 0.92 | 0.11 | 1.10(0.92-1.32) | 0.30 | 0.26 | 1.11(0.93-1.32) | 0.25 | 0.42 | 1.00(0.87-1.14) | 0.98 | 0.23 |

TB=Tuberculosis, P*_Eff_* =P value of pooled effect, P*_Het_* =P value of heterogeneity test.
