# Supplementary material for: Interleukin-10 Promoter Gene Polymorphisms and Susceptibility to Tuberculosis: A Meta-Analysis
Source: PLoS One. 2015 Jun 1;10(6):e0127496. doi: 10.1371/journal.pone.0127496 (PMC4452516; doi:10.1371/journal.pone.0127496)
Supplement: S3 Table — (DOCX) [file pone.0127496.s006.docx]

**Table S3. Meta-analysis of the association between the IL-10 -1082 G/A polymorphism and TB** **for fixed effect model.**

|  | No. | A vs G | | | AA vs GG | | | AA+AG vs GG | | | AA vs AG+GG | | |
| --- | --- | --- | --- | --- | --- | --- | --- | --- | --- | --- | --- | --- | --- |
| Population |  | OR(95% CI) | *P_Eff_* | P_Het_ | OR(95% CI) | *P_Eff_* | P_Het_ | OR(95% CI) | *P_Eff_* | P_Het_ | OR(95% CI) | *P_Eff_* | P_Het_ |
| Overall | 22 | 1.00(0.94-1.06) | 0.88 | <0.00001 | 0.96(0.83-1.11) | 0.62 | <0.00001 | 0.95(0.84-1.09) | 0.47 | <0.00001 | 1.01(0.94-1.09) | 0.83 | <0.00001 |
| Subgroup by ethnicity | | | | | | | | | | | | | |
| Asian | 12 | 1.07(0.82-1.38) | 0.63 | <0.0001 | 0.91(0.66-1.26) | 0.57 | <0.0001 | 0.91(0.71-1.16) | 0.44 | <0.00001 | 1.13(1.00-1.29) | 0.06 | <0.00001 |
| European | 4 | 0.68(0.53-0.86) | 0.001 | 0.004 | 0.54(0.33-0.88) | 0.01 | 0.008 | 0.62(0.40-0.96) | 0.03 | 0.08 | 0.61(0.44-0.86) | 0.004 | 0.003 |
| African | 4 | 1.02(0.94-1.10) | 0.67 | 0.17 | 1.11(0.93-1.33) | 0.23 | 0.36 | 1.11(0.93-1.32) | 0.24 | 0.42 | 0.99(0.90-1.10) | 0.88 | 0.23 |

TB=Tuberculosis, P*_Eff_* =P value of pooled effect, P*_Het_* =P value of heterogeneity test.
