# Supplementary material for: Interleukin-10 Promoter Gene Polymorphisms and Susceptibility to Tuberculosis: A Meta-Analysis
Source: PLoS One. 2015 Jun 1;10(6):e0127496. doi: 10.1371/journal.pone.0127496 (PMC4452516; doi:10.1371/journal.pone.0127496)
Supplement: S4 Table — (DOCX) [file pone.0127496.s007.docx]

**Table S4. Meta-analysis of the association between the IL-10 -819C/T polymorphism and TB for random effect model.**

|  | No. | T vs C | | | TT vs CC | | | CT+TT vs CC | | | TT vs CT+CC | | |  |
| --- | --- | --- | --- | --- | --- | --- | --- | --- | --- | --- | --- | --- | --- | --- |
| Population |  | OR(95% CI) | *P_Eff_* | P_Het_ | OR(95% CI) | *P_Eff_* | P_Het_ | OR(95% CI) | *P_Eff_* | P_Het_ | OR(95% CI) | *P_Eff_* | P_Het_ |  |
| Overall | 17 | 1.03(0.94-1.12) | 0.57 | 0.04 | 1.03(0.88-1.22) | 0.69 | 0.16 | 1.05(0.92-1.19) | 0.46 | 0.09 | 1.01(0.92-1.11) | 0.83 | 0.21 |  |
| Subgroup by ethnicity | | | | | | | | | | | | | |  |
| Asian | 7 | 1.17(1.05-1.29) | 0.003 | 0.49 | 1.37(1.09-1.73) | 0.006 | 0.67 | 1.33(1.09-1.64) | 0.005 | 0.70 | 1.16(0.99-1.37) | 0.07 | 0.32 |  |
| European | 4 | 0.77(0.52-1.15) | 0.20 | 0.07 | 0.62(0.30-1.29) | 0.20 | 0.24 | 0.78(0.50-1.24) | 0.30 | 0.13 | 0.69(0.38-1.26) | 0.23 | 0.36 |  |
| African | 5 | 0.97(0.90-1.04) | 0.33 | 0.64 | 0.91(0.79-1.06) | 0.22 | 0.89 | 0.98(0.87-1.10) | 0.38 | 0.32 | 0.91(0.80-1.04) | 0.16 | 0.87 |  |

TB=Tuberculosis, P*_Eff_* =P value of pooled effect, P*_Het_* =P value of heterogeneity test.
