# Supplementary material for: Interleukin-10 Promoter Gene Polymorphisms and Susceptibility to Tuberculosis: A Meta-Analysis
Source: PLoS One. 2015 Jun 1;10(6):e0127496. doi: 10.1371/journal.pone.0127496 (PMC4452516; doi:10.1371/journal.pone.0127496)
Supplement: S5 Table — (DOCX) [file pone.0127496.s008.docx]

**Table S5. Meta-analysis of the association between the IL-10 -819C/T polymorphism and TB** **for fixed effect model.**

|  | No. | T vs C | | | TT vs CC | | | CT+TT vs CC | | | TT vs CT+CC | | |  |
| --- | --- | --- | --- | --- | --- | --- | --- | --- | --- | --- | --- | --- | --- | --- |
| Population |  | OR(95% CI) | *P_Eff_* | P_Het_ | OR(95% CI) | *P_Eff_* | P_Het_ | OR(95% CI) | *P_Eff_* | P_Het_ | OR(95% CI) | *P_Eff_* | P_Het_ |  |
| Overall | 17 | 1.02(0.96-1.07) | 0.58 | 0.03 | 1.01(0.89-1.14) | 0.90 | 0.16 | 1.03(0.94-1.12) | 0.53 | 0.09 | 1.01(0.92-1.11) | 0.80 | 0.18 |  |
| Subgroup by ethnicity | | | | | | | | | | | | | |  |
| Asian | 7 | 1.17(1.05-1.29) | 0.003 | 0.49 | 1.37(1.09-1.72) | 0.006 | 0.67 | 1.33(1.09-1.63) | 0.006 | 0.70 | 1.17(1.02-1.35) | 0.03 | 0.32 |  |
| European | 4 | 0.84(0.66-1.07) | 0.15 | 0.07 | 0.61(0.34-1.11) | 0.11 | 0.24 | 0.85(0.62-1.16) | 0.30 | 0.16 | 0.66(0.37-1.17) | 0.15 | 0.36 |  |
| African | 5 | 0.97(0.90-1.04) | 0.33 | 0.64 | 0.91(0.79-1.06) | 0.22 | 0.89 | 0.98(0.89-1.09) | 0.74 | 0.32 | 0.91(0.80-1.04) | 0.16 | 0.87 |  |

TB=Tuberculosis, P*_Eff_* =P value of pooled effect, P*_Het_* =P value of heterogeneity test.
