# Supplementary material for: Interleukin-10 Promoter Gene Polymorphisms and Susceptibility to Tuberculosis: A Meta-Analysis
Source: PLoS One. 2015 Jun 1;10(6):e0127496. doi: 10.1371/journal.pone.0127496 (PMC4452516; doi:10.1371/journal.pone.0127496)
Supplement: S6 Table — (DOCX) [file pone.0127496.s009.docx]

**Table S6. Meta-analysis of the association between the IL-10 -592A/C polymorphism and TB for random effect model.**

| Population | No. | A vs C | | | AA vs CC | | | AA vs AC+CC | | | AA+AC vs CC | | |  |
| --- | --- | --- | --- | --- | --- | --- | --- | --- | --- | --- | --- | --- | --- | --- |
|  |  | OR(95% CI) | *P_Eff_* | P_Het_ | OR(95% CI) | *P_Eff_* | P_Het_ | OR(95% CI) | *P_Eff_* | P_Het_ | OR(95% CI) | *P_Eff_* | P_Het_ |  |
| Overall | 16 | 0.99(0.87-1.12) | 0.84 | <0.0001 | 0.99(0.79-1.25) | 0.96 | 0.002 | 1.02(0.86-1.21) | 0.79 | 0.006 | 0.97(0.82-1.15) | 0.70 | 0.002 |  |
| Subgroup by ethnicity | | | | | | | | | | | | | |  |
| Asian | 6 | 1.22(0.96-1.55) | 0.11 | 0.0004 | 1.50(0.89-2.53) | 0.13 | 0.001 | 1.26(0.91-1.74) | 0.17 | 0.002 | 1.32(0.94-1.85) | 0.11 | 0.04 |  |
| European | 4 | 0.73(0.52-1.03) | 0.07 | 0.16 | 0.55(0.30-1.01) | 0.05 | 0.38 | 0.61(0.34-1.08) | 0.09 | 0.50 | 0.73(0.49-1.10) | 0.13 | 0.21 |  |
| African | 4 | 0.96(0.88-1.05) | 0.39 | 0.48 | 0.92(0.77-1.10) | 0.36 | 0.81 | 0.91(0.77-1.07) | 0.25 | 0.84 | 0.96(0.80-1.14) | 0.62 | 0.18 |  |

TB=Tuberculosis, P*_Eff_* =P value of pooled effect, P*_Het_* =P value of heterogeneity test.
