# Supplementary material for: Interleukin-10 Promoter Gene Polymorphisms and Susceptibility to Tuberculosis: A Meta-Analysis
Source: PLoS One. 2015 Jun 1;10(6):e0127496. doi: 10.1371/journal.pone.0127496 (PMC4452516; doi:10.1371/journal.pone.0127496)
Supplement: S8 Table — (DOCX) [file pone.0127496.s011.docx]

**Table S8. Meta-analysis of the association between the IL-10 promoter haplotype (-1082G/A, 819C/T, 592A/C) and TB for random effect model.**

| Population | No. | GCC vs others | | | n | ACC vs others | | | n | ATA vs others | | |
| --- | --- | --- | --- | --- | --- | --- | --- | --- | --- | --- | --- | --- |
|  |  | OR(95% CI) | *P_Eff_* | P_Het_ |  | OR(95% CI) | *P_Eff_* | P_Het_ |  | OR(95% CI) | *P_Eff_* | P_Het_ |
| Overall | 6 | 1.42(1.02-1.97) | 0.04 | 0.009 | 6 | 0.85(0.68-1.05) | 0.14 | 0.02 | 5 | 0.90(0.78-1.04) | 0.17 | 0.66 |
| Subgroup by ethnicity | | | | | | | | | | | | |
| Asian | 3 | 1.30(0.93-1.82) | 0.12 | 0.70 | 2 | 0.85(0.72-1.00) | 0.04 | 0.78 | 2 | 1.07(0.81-1.41) | 0.63 | 0.99 |
| European | 2 | 2.15(1.53-3.01) | <0.0001 | 0.80 | 2 | 0.60(0.43-0.83) | 0.002 | 0.39 | 2 | 0.78(0.56-1.09) | 0.15 | 0.77 |
|  |  |  |  |  |  |  |  |  |  |  |  |  |

TB=Tuberculosis, P*_Eff_* =P value of pooled effect, P*_Het_* =P value of heterogeneity test.
